# TAp73alpha protects small cell lung carcinoma cells from caspase-2 induced mitochondrial mediated apoptotic cell death

**DOI:** 10.18632/oncotarget.391

**Published:** 2011-12-22

**Authors:** Naveen Muppani, Ulrika Nyman, Bertrand Joseph

**Affiliations:** ^1^ Department of Oncology-Pathology, Cancer Centrum Karolinska, Karolinska Institutet, 171 76 Stockholm, Sweden; ^2^Present address: Ludwig Institute for Cancer Research, 171 77 Stockholm, Sweden

**Keywords:** p73, caspase-2, apoptosis, cancer

## Abstract

Caspase-2 is ubiquitously expressed and the most evolutionarily conserved mammalian caspase. It can be activated by a range of death stimuli prior to Bax activation and the occurrence of apoptotic mitochondrial dysfunctions. Caspase-2 has also been reported to exert tumour suppressor function *in vivo*. The full length TAp73alpha isoform is found up-regulated in various tumour types, and is reported in a cell-type specific manner to repress drug-induced apoptosis. Here, we report that TAp73alpha represses caspase-2 enzymatic activity and by this means reduce caspase-2 induced Bax activation, loss of mitochondrial transmembrane potential and resulting apoptosis. The inhibitory effect on caspase-2 requires the presence of the DNA binding domain and SAM domain region of TAp73alpha. In conclusion, the ability of TAp73alpha to act as an inhibitor of caspase-2-induced cell death together with its up-regulation in certain tumour types strengthen the potential oncogenic activities for this protein.

## INTRODUCTION

The transcription factor p73 is a multi-functional protein that belongs to the p53 family of proteins. Indeed, p73 is able to regulate cell cycle arrest [1], apoptosis [2], differentiation [3] and development [4]. The p73 protein contains a NH2-terminal transactivation (TA) domain, a highly conserved core DNA-binding domain (DBD), and a COOH-terminal oligomerization domain [5-6]. The *P73* gene has two alternative promoters (p1 and p2) and gives rise to a vast number of isoforms due to the use of these promoters and from alternative splicing at the COOH-terminal region. The two alternative promoters located at the NH2-terminal region generate either transactivation (TA) competent or TA deficient (deltaN) primary transcripts of p73. Alternative splicing of each of these primary p73 transcripts can theoretically generate nine different COOH-terminal isoform variants (alpha, beta, gamma, delta, epsilon, theta, zeta, eta and eta1) [7]. Among these isoforms, TAp73alpha and TAp73beta are the two main expressed isoforms in human cells. TAp73alpha is the longest isoform, comprise an extended COOH-terminus including a sterile alpha motif (SAM) region. TAp73beta is a shorter isoform, and lacks the extreme COOH-terminal region and SAM domain [8]. p73 and its family member p53 share both structural and functional properties [9-11]. Nevertheless, some key differences between the two proteins do exist, for example, the novel structural protein NSP 5a3a can induce apoptosis via p73, independent of p53 [12]. Moreover, *P73* gene mutations are seldom noticed in tumours as compared with *P53* gene mutations [13]. However, mice selectively devoid of the TAp73 isoforms (TAp73 null mice) do show increased incidence of spontaneous tumors [14]. On the other hand, increased TAp73alpha expression levels has been noticed in certain cancers like, cervical cancer [7], medulloblastoma [15], B-cell chronic lymphocytic leukaemia [16], ovarian carcinomas [17], gastric adenocarcinoma [18], bladder cancer [19] and thyroid cancer [20]. In some tumour cell lines, increased levels of TAp73alpha were detected after various drug treatments, e.g. etoposide (VP16; Vepesid®), and camptothecin [21]. We previously reported that TAp73alpha inhibits drug (*i.e.* VP16, cisplatin and staurosporine)-induced apoptosis in small cell lung carcinoma (SCLC) cells. The anti-apoptotic effect is exerted upstream of mitochondrial outer membrane permeabilization (MOMP), at the level of Bax activation [22].

Interestingly, caspase-2, one of the ubiquitously expressed and the most evolutionarily conserved mammalian caspase, can be activated before the MOMP that occurs during apoptosis induced by a range of death stimuli [23-33]. In fact, caspase-2 has long been recognized as an important protein in the regulation of apoptosis [30, 34]. Caspase-2 contains a caspase activation and recruitment domain (CARD), and biochemical studies indicate that the primary event required for caspase-2 activation is CARD-dependent dimerization. Recruitment of caspase-2 to activation complexes, such as the PIDDosome, induce caspase-2 proximity and hence, its activation [35-36]. Worth to notice is that caspase-2 over-expression *per se* leads to CARD-mediated dimerization, enzyme activation and induction of apoptotic cell death [35, 37]. Active caspase-2 was found to engage mitochondria by directly cleaving full-length Bid to activated tBid and promoting Bax translocation to the mitochondria [27, 30, 38].

Caspases-3, -7 and -9 have been shown to be susceptible to inhibition by members of the inhibitors of apoptosis (IAP) family [39]. Despite the fact that caspase-2 was the second caspase to be cloned in 1992, to date no natural caspase-2 inhibitors have been reported (with the exception of some baculoviral proteins, e.g. p35 and p49) [37]. Since TAp73alpha has been reported to exert its anti-apoptotic effects in SCLC cells upstream of Bax activation, this inspired us to investigate whether TAp73alpha could modulate cell death mediated by caspase-2. In the present study we reveal that TAp73alpha is able to repress caspase-2 induced apoptosis in SCLC NCI-H82 cells, through inhibition of caspase-2 enzymatic activity. Upon TAp73alpha expression, caspase-2 induced Bax activation, loss of mitochondrial membrane potential and consequent cell death were found reduced. Moreover, we report that both the DBD and the SAM domain of TAp73alpha are required for its anti-apoptotic effects on caspase-2 induced cell death.

## RESULTS

### TAp73alpha inhibits apoptosis induced by caspase-2 over-expression in SCLC NCI-H82 cells

**Figure 1:**
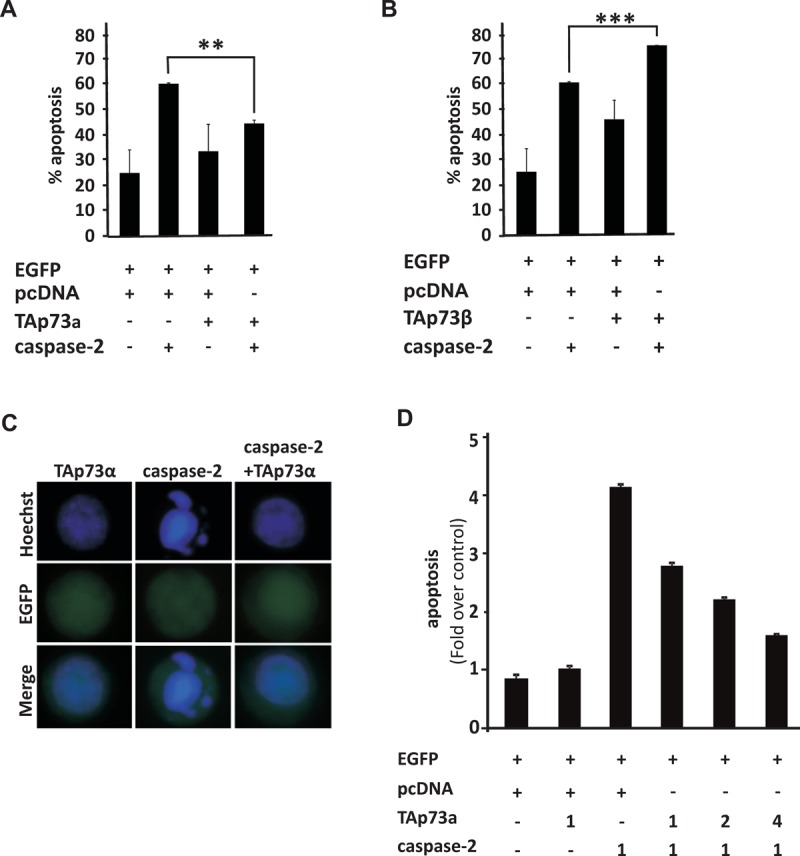
TAp73alpha inhibits apoptosis induced by caspase-2 over-expression in SCLC NCI-H82 cells SCLC NCI-H82 cells (Figure 1A-D) were co-transfected with EGFP together with either an empty (control) vector or plasmids encoding TAp73alpha, TAp73beta and/or caspase-2. Twenty-four hours post-transfection, cells were fixed in 4% PFA, stained with Hoechst and scored under a fluorescence microscope as percentage of EGFP expressing cells displaying condensed or fragmented nuclei. Three hundred EGFP transfected cells were analyzed and each experiment was repeated three times. Figures are mean + SD of three independent experiments, where ***p-value <0.001, **p-value <0.01

Caspase-2 processing and activation occurs rapidly in response to both intrinsic and extrinsic cell death signalling [40-42]. In turn, active caspase-2 promotes Bax translocation to the mitochondria [27]. Moreover, the ability of over-expressed caspase-2 to promote apoptosis is established [34, 43]. We have previously shown that full-length TAp73alpha represses drug-induced apoptosis in SCLC cells, upstream of the mitochondria at the level of Bax activation [22, 44]. To test whether caspase-2 over-expression induces apoptosis in SCLC NCI-H82 cells and whether TAp73alpha can exert a repressive effect on caspase-2 induced cell death, apoptosis assays were performed. NCI-H82 SCLC cells were co-transfected with EGFP together with either an empty (control) vector or plasmids encoding TAp73alpha, TAp73beta and/or caspase-2. Twenty-four hours post-transfection, cells were fixed with 4% PFA, stained with Hoechst and scored under a fluorescence microscope as percentage of EGFP expressing cells displaying apoptotic nuclei. In NCI-H82 cells, caspase-2 expression induced a robust apoptotic cell death (Fig. 1A, 1B and 1D), whereas TAp73alpha expression only caused small increase in apoptosis as compared to the basal level (Fig. 1A and D). Remarkably, co-expression of TAp73alpha together with caspase-2 led to a significant decrease in the percentage of apoptotic cells as compared to cells transfected with caspase-2 alone (Fig. 1A, 1C and D). On the other hand, co-expression with TAp73beta significantly promoted caspase-2 induced apoptosis (Fig. 1B). Increasing the ratio of TAp73alpha to caspase-2 expression decreased caspase-2 induced apoptosis in a dose dependent manner (Fig. 1D). Hence, caspase-2 over-expression *per se* can induce apoptosis in SCLC NCI-H82 cells, an event that can be prevented by the simultaneous co-expression of anti-apoptotic TAp73alpha.

### TAp73alpha expression inhibits caspase-2 activity

**Figure 2:**
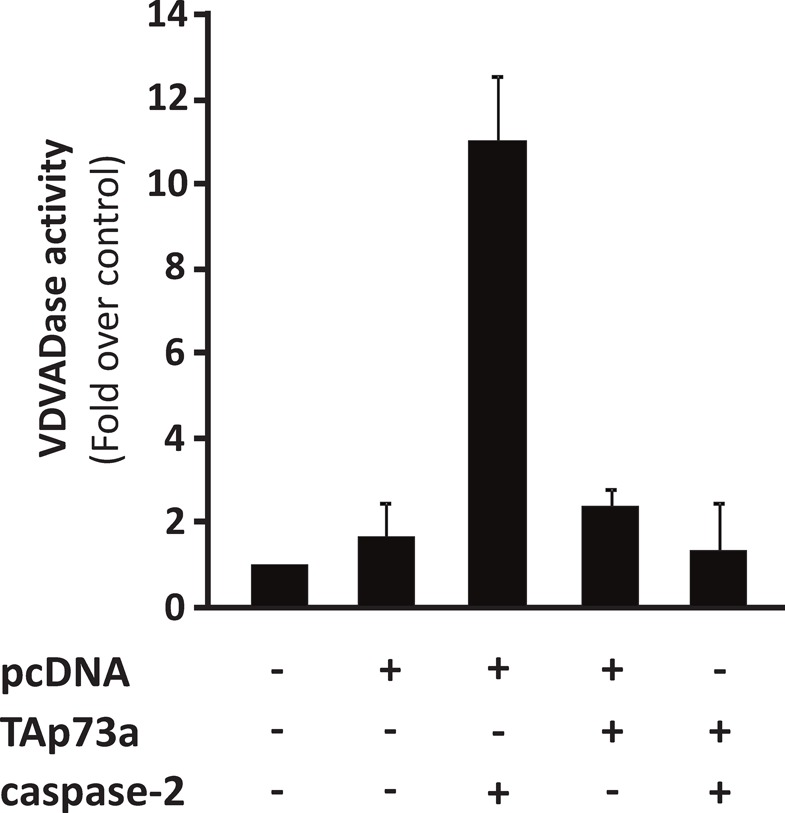
TAp73alpha expression inhibits caspase-2 activity SCLC NCI-H82 cells (Figure 2) were transfected with TAp73alpha, caspase-2 and/or empty expression vectors, and caspase-2 activity was monitored. The enzyme activity of caspase-2 (VDVADase activity) was evaluated as the release of AMC from the substrate Ac-VDVAD-AMC (Ac-Val-Asp-Val-Ala-Asp-7-amino-4-methylcoumarin). The data represents three independent experiments.

Thereafter, we investigated whether the protective effect of TAp73alpha on caspase-2 induced apoptosis could be mediated by direct physical protein-protein interaction. Hence, co-immunoprecipitation experiments were performed, but no physical interaction between these two proteins could be detected. Despite the apparent lack of protein-protein interaction, we speculated that TAp73alpha might affect the actual enzymatic activity of caspase-2. NCI-H82 cells were transfected with TAp73alpha, caspase-2 and/or empty expression vectors, and caspase-2 activity was monitored. The enzyme activity of caspase-2 (VDVADase activity) was evaluated as the release of AMC from the substrate Ac-VDVAD-AMC (Ac-Val-Asp-Val-Ala-Asp-7-amino-4-methylcoumarin) [28]. As previously reported in other cell types, caspase-2 over-expression lead to increased VDVADase activity in NCI-H82 cells (Fig. 2). TAp73alpha over-expression caused a small increase in caspase-2 activity, compared to the mock vector transfected cells. Interestingly, co-expression of TAp73alpha with caspase-2 significantly prevented caspase-2 induced VDVADase activity in NCI-H82 cells (Fig. 2). Altogether these data indicate that TAp73alpha is able to inhibit the enzyme activity of caspase-2 in SCLC NCI-H82 cells.

### TAp73alpha prevents caspase-2 induced mitochondrial dysfunction in SCLC NCI-H82 cells

Fully processed and active caspase-2 can permeabilize the outer mitochondrial membrane and cause cytochrome *c release* [33, 45], which activates the apoptotic cascade [28]. We have shown that TAp73alpha is able to prevent VP16-induced loss of mitochondrial membrane potential in SCLC cells [22, 44]. To explore whether the protective effect of TAp73alpha over-expression on caspase-2 induced apoptosis involve repression of mitochondrial dysfunction, the mitochondrial transmembrane potential was investigated.

**Figure 3:**
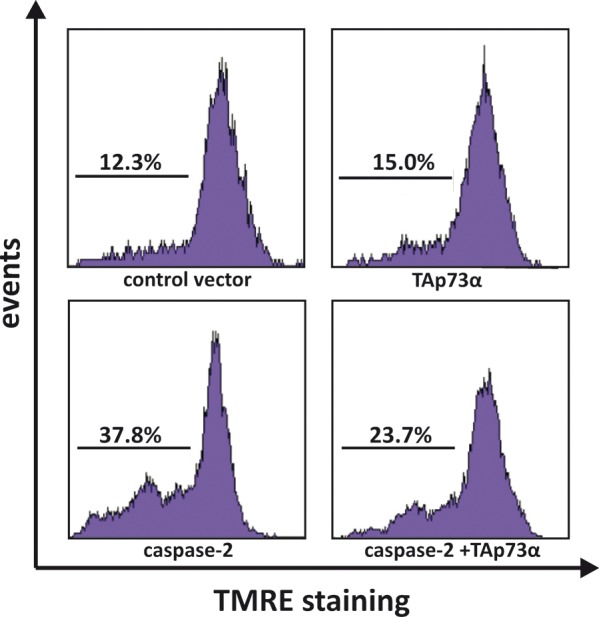
TAp73alpha prevents caspase-2 induced mitochondrial dysfunction in SCLC NCI-H82 cells SCLC NCI-H82 cells (Figure 3) were co-transfected with TAp73alpha, caspase-2 and/or empty expression vectors. Twenty-four hours post-transfection, cells were stained with TMRE and loss of mitochondrial trans-membrane potential (TMRE-negative cells) was assayed by scoring of EGFP transfected cells without TMRE by FACS analysis. Images are representatives of three independent experiments.

NCI-H82 cells were co-transfected with EGFP together with either an empty (control) vector or plasmids encoding TAp73alpha, and/or caspase-2. Twenty-four hours post-transfection, cells were stained with TMRE and then assessed for the loss of mitochondrial transmembrane potential (TMRE-negative cells) by scoring EGFP expressing cells by FACS analysis. In NCI-H82 cells, caspase-2 over-expression provoked a noteworthy loss of mitochondrial transmembrane potential (37.8% cells with loss of mitochondrial membrane potential), whereas TAp73alpha expression showed limited effect as compared to control (15% vs. 12.3% cells with loss of mitochondrial membrane potential) (Fig. 3). Interestingly, caspase-2 induced loss of mitochondrial transmembrane potential was reduced when cells were co-transfected with TAp73alpha (from 37.8% to 23.7% cells with loss of mitochondrial membrane potential) (Fig. 3). Altogether these data indicate that TAp73alpha inhibits the caspase-2 induced loss of mitochondrial transmembrane potential in SCLC NCI-H82 cells.

### TAp73alpha counteracts caspase-2 induced Bax activation in SCLC NCI-H82 cells

**Figure 4:**
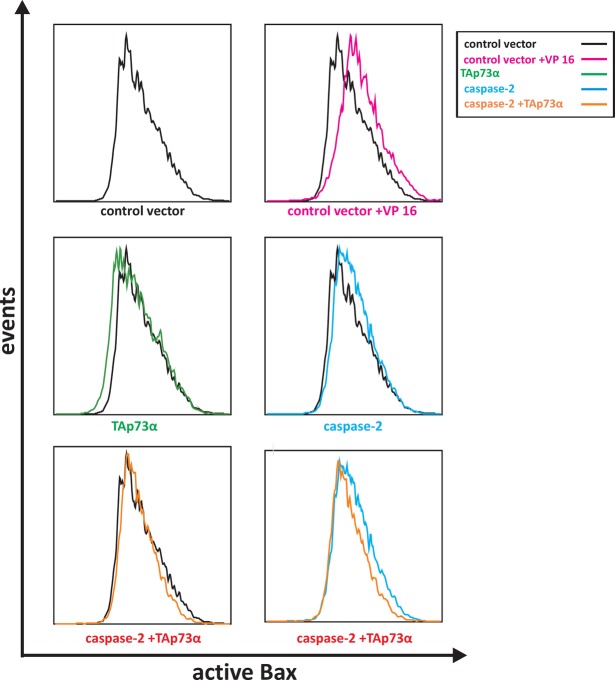
TAp73alpha counteracts caspase-2 induced Bax activation in SCLC NCI-H82 cells SCLC NCI-H82 cells (Figure 4) were co-transfected with EGFP and plasmids encoding TAp73alpha, caspase-2 and/or empty expression vectors. Twenty-four hours post-transfection, SCLC NCI-H82 cells were collected, fixed in 4% PFA and incubated with the 6A7 anti-Bax antibody at 4°C, overnight, followed by secondary Alexa Fluor 635-conjugated antibody. For each sample 10,000 cells were sorted for green (EGFP) fluorescence using BD LSR II flow cytometer, and assessed for intensity of active Bax staining.

It has previously been shown that caspase-2 is essential for the integration of cytosolic Bax into the outer mitochondrial membrane, an event that is important for subsequent activation of Bax [46]. We have previously shown that TAp73alpha is able to prevent Bax activation upon VP16 treatment [22, 44]. Thus, we decided to explore whether the protective effect of TAp73alpha over-expression on caspase-2 induced apoptosis is due to repression of Bax activation. To investigate how caspase-2 and TAp73alpha regulate Bax activation, we took advantage of the 6A7 anti-Bax antibody, which recognizes the membrane-bound active form of Bax [47]. NCI-H82 cells were co-transfected with EGFP and plasmids encoding TAp73alpha, caspase-2 and/or empty expression vectors. Twenty-four hours post-transfection, NCI-H82 cells were collected and probed with the 6A7 anti-Bax antibody and subsequently incubated with Alexa Fluor 635-conjugated secondary antibody. The samples were analyzed for increased fluorescence by FACS to assess Bax activation. As shown in figure 4, over-expression of caspase-2 in NCI-H82 cells leads to Bax conformational changes (Fig. 4, middle, right histogram). Co-expression of TAp73alpha resulted in a decrease of caspase-2-induced Bax activation (Fig. 4, bottom, right histogram). Taken together, in SCLC NCI-H82 cells, TAp73alpha seems to counteract the effect of caspase-2 on Bax, and consequently inhibit the mitochondrial cell death pathway activated upon Bax conformational changes.

### TAp73alpha DBD and SAM domain are required for inhibition of caspase-2 induced apoptosis

**Figure 5:**
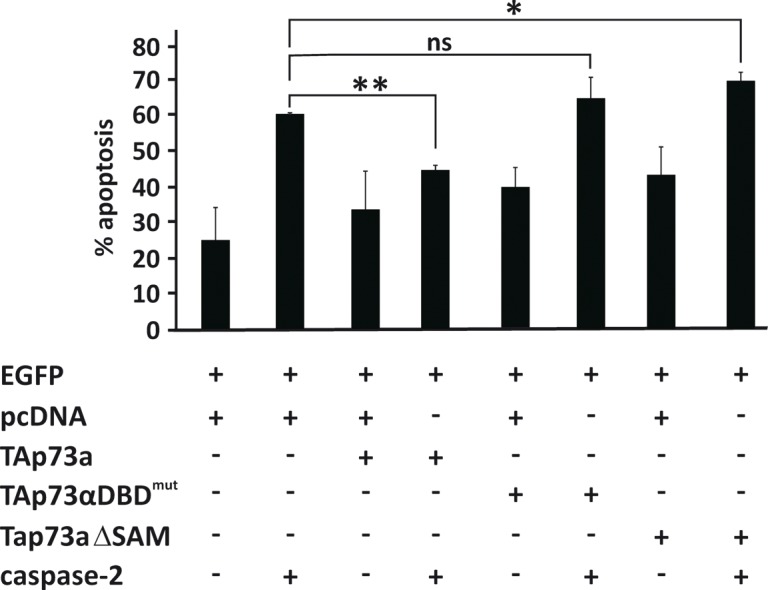
The DNA binding domain and SAM domain are required for TAp73alpha inhibition of caspase-2 induced apoptosis SCLC NCI-H82 cells (Figure 5) were co-transfected with EGFP together with either an empty (control) vector, TAp73alpha or plasmids encoding different mutant forms of TAp73alpha (DBDmutp73alpha/p73alphaSAMdel), and/or caspase-2. Twenty-four hours post-transfection, cells were fixed, stained with Hoechst and scored under a fluorescence microscope as percentage of EGFP expressing cells displaying condensed or fragmented nuclei. Three hundred EGFP transfected cells were analyzed and each experiment was repeated three times. Figures are mean + SD of three independent experiments, where *p <0.05.

We previously characterized which domains of TAp73alpha are essential for the repression of VP16-induced apoptosis in SCLC NCI-H82 cells. The DBD and the unique carboxyl terminus SAM domain of p73alpha were both found to be required for its anti-apoptotic function [22]. To detect the domains that were required for TAp73alpha inhibitory effect on caspase-2 induced apoptosis, NCI-H82 cells were co-transfected with EGFP together with either an empty (control) vector or plasmids encoding different mutant forms of TAp73alpha and/or caspase-2 and apoptosis assay was performed. In the context of caspase-2 induced cell death, the TAp73alphaDBD mutant construct with a double point mutation in the DNA binding domain [48] was not able to modulate apoptosis induced by caspase-2 over-expression (Fig. 5). The deletion construct of TAp73alpha lacking the SAM domain was also unable to inhibit caspase-2 induced apoptosis (Fig. 5). In fact, over-expression of the TAp73alphaSAMdel mutant potentiated caspase-2 induced apoptosis in NCI-H82 SCLC cells, in a manner similar to that of TAp73beta. These data suggest that both the DBD and the SAM domain of TAp73alpha are involved in the inhibition of caspase-2 induced cell death.

## DISCUSSION

Although both TAp73alpha and TAp73beta can induce cell cycle arrest and apoptosis upon drug treatment, TAp73alpha expression is increased in a range of tumours and has been shown to confer resistance of some tumour cells towards drug-treatment [22, 44, 49]. Previously, we have shown that TAp73alpha can repress drug-induced apoptosis in a cell-type specific manner. We demonstrated this effect to be exerted upstream of the mitochondria, at the level of Bax activation [22, 44]. Here, we provide an additional mechanism in support of the anti-apoptotic effect of TAp73alpha. Caspase-2 activation occurs rapidly in response to a wide range of death stimuli and is associated with Bax activation and mitochondrial dysfunction, suggesting that it may have a general role in apoptosis [40-42]. In this study we show that the TAp73alpha, but not the TAp73beta isoform, is able to repress caspase-2 induced cell death in SCLC NCI-H82 cells. In these cells, we found that TAp73alpha inhibit caspase-2 enzymatic activity and thereby prevent caspase-2 induced Bax activation and subsequent loss of mitochondrial transmembrane potential.

Some of the functional differences between TAp73 isoforms, e.g. TAp73alpha and TAp73beta, are likely to be explained by the specific interactions of TAp73alpha unique domains, i.e. SAM domain and extreme carboxy-terminus, with other proteins. Specific p63-regulated micro-RNAs have been shown to regulate TAp73 levels in general [50], but the receptor for activated kinase 1 (RACK1) only interacts with TAp73alpha carboxy-terminus, ultimately resulting in reduced transcriptional activity and inhibition of TAp73alpha-induced apoptosis [51]. Due to its extended carboxy-terminus, TAp73alpha, but not TAp73beta, can be covalently modified by SUMO-1. Sumoylation at the COOH-terminal lysine 627 leads to a more rapid degradation of TAp73alpha by the proteasome [52]. Here, we characterized the structure requirement for the repressive effect of TAp73alpha on caspase-2 induced cell death in SCLC NCI-H82 cells and found that the DNA binding domain as well as the unique SAM domain of TAp73alpha are required for the suppression of caspase-2 induced cell death.

Recent investigations suggests that caspase-2 may have additional roles in DNA damage response and cell cycle regulation [23]. Indeed, several recent publications provide evidences that caspase-2 can function as a regulator of cell cycle checkpoints, and in DNA repair [53-54].

Furthermore, in an *E mu-Myc* mouse model of lymphomagenesis, loss of caspase-2 results in an increased ability of cells to acquire a transformed phenotype and become malignant, indicating that caspase-2 functions as a tumour suppressor [55]. Whether TAp73alpha can also alter these additional caspase-2 functions remain to be established. Nevertheless, considering that TAp73alpha overexpression is associated with resistance to treatment with DNA-damaging agents in human ovarian cancer and SCLC cell lines [22, 49], this possibility cannot be excluded.

As mentioned earlier, caspases-3, -7 and -9 are susceptible to inhibition by members of the inhibitors of apoptosis (IAP) family, and with the exception of some baculoviral proteins (e.g. p35 and p49), no natural caspase-2 inhibitors have been reported. Here we report that TAp73alpha, a member of p53 family of proteins, inhibits caspase-2 enzymatic activity and protects SCLC NCI-H82 cells from caspase-2 mediated mitochondrial cell death. Moreover, we conclude that the anti-apoptotic effect of TAp73alpha requires the presence of a functional DNA binding domain and SAM domain, and operates at the initiation phase of apoptosis, upstream of Bax activation and loss of the mitochondrial transmembrane potential. Caspase-2 activation occurs rapidly in response to a range of death stimuli and is associated with both Bax activation and mitochondrial dysfunction, suggesting that it may have a general role in apoptosis [40-42]. Thus, the ability of TAp73alpha to inhibit caspase-2 induced cell death and its up-regulation in certain tumour types [15-20, 56] uncover new potential oncogenic activities for this protein. NF-kappaB, a transcription factor which exhibits both pro- and anti-apoptotic characters [57], was suggested to have a Jekyll and Hyde behaviour in a cell context dependent manner [58]. This behaviour also seems to apply to TAp73alpha, a behaviour we obviously need to understand to make the most use of drugs and cancer treatments in general.

## MATERIALS AND METHODS

### Plasmids

Expression vectors encoding human TAp73alpha and TAp73beta were kind gifts from Dr. G. Melino and have been described [59-60]. The TAp73alphaDBD mutant having amino acid substitutions at positions 268 and 300 on the DNA-binding domain was a generous gift from Dr. S. Deb [48]. Carboxyl-terminus deleted TAp73alpha mutant lacking the SAM domain, TAp73alphaSAMdel was a kind gift from Dr M. Hijikata [61]. Dr. S. Kumar kindly provided us with plasmid encoding caspase-2 [35]. Enhanced green fluorescent protein (EGFP) plasmid was from Clontech. The empty pcDNA-3.1 vector was used as control (mock).

### Cell Culture, transfection and treatments

The human small cell lung carcinoma NCI-H82 (ATCC HTB-175) cell line was used in this study. The NCI-H82 cells produce an abnormally sized p53 mRNA (3.7 kb) and do not show detectable levels of p53 protein. Cells were cultured at 37°C, 5% CO_2_ in RPMI 1640 medium, supplemented with 10% heat-inactivated fetal calf serum, 2 mM L-glutamine, penicillin (100 units/ml), and streptomycin (100 mg/ml). Twenty-four hours after setting in culture dishes with fresh medium, cells were transfected using Lipofectamine 2000 (Invitrogen) according to the manufacturer's protocol. If not stated otherwise, the co-transfection ratio of TAp73alpha to caspase-2 was always kept at 2:1. When indicated, H82 cells were treated with 10 uM etoposide (VP16; Bristol-Myers) for 24 hours. The cell density was kept at levels allowing exponential growth.

### Flow cytometric analysis of Bax activation

Bax-associated conformational changes were assessed as previously described [44]. Briefly, one day after co-transfection of EGFP and plasmids encoding TAp73alpha, caspase-2 and/or empty (control) vector, H82 cells were harvested, fixed in 4% PFA and blocked/permeabilized in PBS with 10 mM HEPES, 0.3% Triton X-100 and 3% BSA. Subsequently, cells were incubated with an antibody recognizing N-terminal epitopes of Bax (clone 6A7; BD Biosciences) at 4°C, overnight, followed by secondary Alexa Fluor 635-conjugated antibody (Invitrogen) at room temperature, for 30 minutes. Cells were re-suspended in PBS. For each sample 10,000 cells were sorted for green (EGFP) fluorescence using BD LSR II flow cytometer, and assessed for intensity of Bax staining using FlowJo software version 9.4.3 (Tree Star, Ashland, OR).

### Flow cytometric analysis of mitochondrial depolarization

Cytofluorometric analysis of mitochondrial membrane depolarization was assessed by uptake of tetramethylrhodamine ethyl esters (TMRE; Molecular Probes/Invitrogen), a lipophilic, cationic fluorochrome dye that is only taken up by mitochondria having an intact electrochemical gradient. H82 cells were co-transfected with EGFP and plasmids encoding TAp73alpha, caspase-2 and/or empty (control) vector. Following TMRE exposure (added 30 min before harvesting to a final concentration of 25 nM), cells were centrifuged and re-suspended in TMRE- containing PBS. Analysis was carried out on 10,000 gated EGFP-expressing cells using a FACSCalibur flow cytometer equipped with CellQuest software (BD Biosciences).

### Detection of Apoptosis

H82 cells were co-transfected with EGFP and plasmids encoding TAp73 isoforms/mutants, caspase-2 and/or empty (control) vector. One day after transfections, cells were fixed with 4% PFA, and nuclei were counterstained using Hoechst 33342 (Molecular Probes/Invitrogen). Number of apoptotic cells was scored under a fluorescence microscope (Zeiss Axioplan 2 Imaging microscope system) by assessing the percentage of EGFP-positive cells displaying apoptotic nuclear morphology (fragmented or condensed nuclei).

### Measurement of caspase-2 activity

H82 cells were transfected with TAp73alpha, caspase-2 and/or empty expression vectors. After 24 hours of transfection, cells were collected, washed in PBS and pellets frozen in eppendorf tubes on liquid nitrogen. After thawing on ice, pellets were re-suspended in 50 ul PBS, and 25 ul of cell lysate transferred to a microtiter plate together with 50 uL of caspase-2 assay buffer (0.1M MES pH 6.5, 0.0001% NP40, 10% PEG, 0.1% CHAPS, 5 mM DTT, 50 uM Ac-VDVAD-AMC). Cleavage of the fluorogenic peptide substrate, Ac-VDVAD-AMC (Ac-Val-Asp-Val-Ala-Asp-7-amino-4-methylcoumarin) was monitored by liberation of AMC in a Fluoroscan II plate reader (Thermo Electron Co., Waltham, MA, USA) by using 355 nM excitation and 460 nM emission wavelengths. Data from triplicate samples were used in each experiment.

### Statistical analysis

Statistical analyses were performed using two-tailed, paired students t-test, where ***p-value <0.001, **p-value <0.01 and *p-value <0.05.
